# Supplementary material for: Discovery of a strain-stabilised smectic electronic order in LiFeAs
Source: Nat Commun. 2018 Jul 4;9:2602. doi: 10.1038/s41467-018-04909-y (PMC6031620; doi:10.1038/s41467-018-04909-y)
Supplement: Supplementary file 1 — Supplementary Information [file 41467_2018_4909_MOESM1_ESM.pdf]

Supplementary Information for
Discovery of a strain-stabilised smectic electronic order in LiFeAs

Yim *et al.*

This PDF file includes:

Supplementary Notes 1 and 2

Supplementary Figs. 1 to 7

Supplementary Note 1. Numerical fitting of the strain-voltage dependent spectra using Dynes equations

We have used multiple methods to ensure that the strain dependence of the gap size is robust against the method used to extract the gap size. The gap sizes reported in Fig. 1e have been determined by fitting a Gaussian peak to the coherence peak and using its position. Here, we show the results obtained by fitting a Dynes equation, accounting for the two superconducting gaps and their broadening, to the same set of data. To this end, we have symmetrised the spectra and then numerically fitted Supplementary Eq. (1) to the data.

$$\rho(E) = \sum_{n=1,2} A_n \times \left| \mathrm{Re}\left[\frac{E - i\Gamma_n}{\sqrt{(E - i\Gamma_n)^2 - \Delta_n^2}} \right] \right| + \rho_0 + \rho_1 E^2 \tag{1}$$

In the equation, Δ_1 (Δ_2) and Γ_1 (Γ_2) represent the size and broadening terms of the outer (inner) gap of LiFeAs, $A_{1,2}$ are the prefactors, the last two terms ($\rho_0 + \rho_1 E^2$) account for an offset and a parabolic background, to account for the normal state density of states. By performing numerical fitting to the symmetrised spectra using Supplementary Eq. (1) with the broadening terms Γ_1 and Γ_2 held constant for all spectra (Supplementary Fig. 7), the plot of Δ_{SC} for both the outer and inner SC gaps as a function of voltage applied to the piezo stack as shown in Supplementary Fig. 7 is obtained, demonstrating a dependence of the SC gap size on the strain voltage, consistent with the behaviour shown in Fig. 1e.

Supplementary Note 2. Determination of the spatial periodicity of the CDW order

Supplementary Fig. 2a shows an atomically resolved STM topographic image of the modulated phase on a strained LiFeAs crystal. In the image, the stripes run along the $[110]$ direction, parallel to the direction of the strain applied to the crystal. In the corresponding Fourier transform (FT) image (Supplementary Fig. 2b), apart from the peaks arising from the atomic lattice of the surface layer of LiFeAs, an additional pair of peaks very close to the origin are present. These additional peaks correspond to the stripes in the modulated phase. To extract the spatial periodicity of the modulation along the $[1\bar{1}0]$ in the real-space image (Supplementary Fig. 2a), we have extracted line profiles from the FT image (Supplementary Fig. 2b) along the $[100]$ (red) and the $[1\bar{1}0]$ (blue) direction. As evidenced by the normalised line-cuts in Supplementary Fig. 2c, the peak due to the modulation is positioned at $0.141q_0$. This means that the stripes in the modulated phase have a real space periodicity of $2.7\mathrm{nm}$ along the $[1\bar{1}0]$ direction in real space. While we always observe the modulation with the same periodicity and direction, it does occasionally exhibit defects as the topological defect shown in Supplementary Fig. 3, or distortions of the stripe pattern.

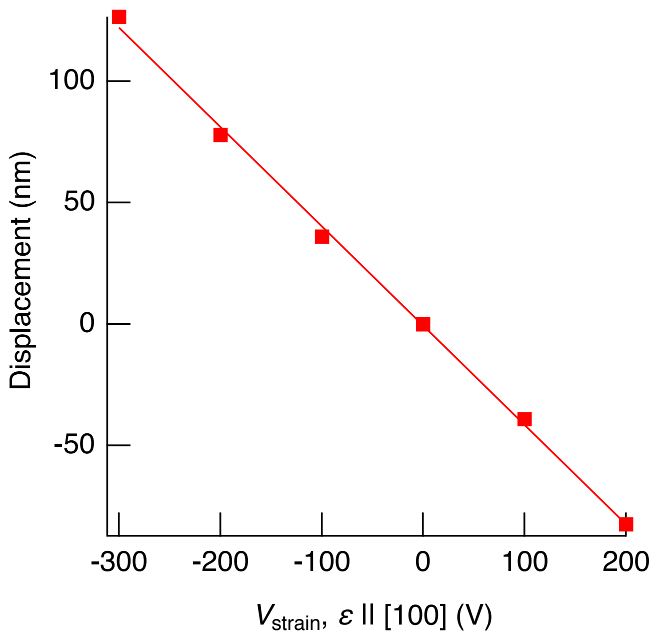

Supplementary Fig. 1: Field-of-view displacement in STM versus voltage applied to piezo. The strain is applied at $18°$ of the [100] direction of the LiFeAs crystal. The solid line is a linear fit to the experimental data, revealing a voltage-induced displacement of $-0.41\mathrm{nm/V}$ along the strain direction.

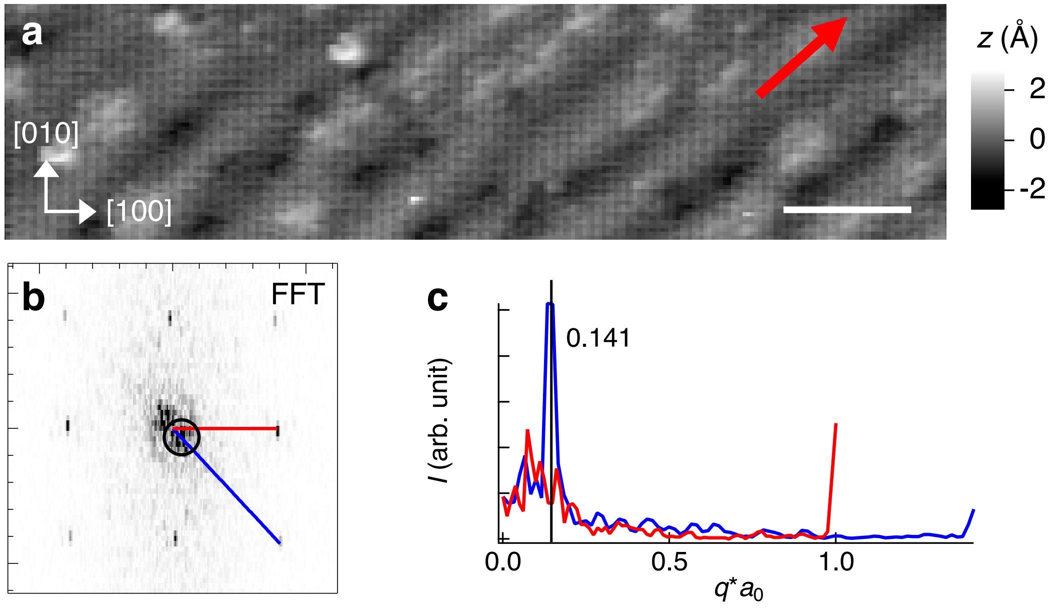

Supplementary Fig. 2: Spatial periodicity of the modulated phase in strained LiFeAs. a, Atomically resolved STM topographic image of the modulated phase ($30 \times 7.5\mathrm{nm}^2$, $V_\mathrm{s} = 10\mathrm{mV}$, $I_\mathrm{s} = 50\mathrm{pA}$). Scale bar = $4\mathrm{nm}$. A red arrow indicates the direction of strain. **b**, Intensity of the Fourier transformation of **a**. An open circle marks one of the peaks which arise from the spatial modulation in the real-space image. **c**, Line-profiles extracted along the blue and red lines in **b**. The x-axis is normalised to the inverse of the lattice constant a_0, where $a_0 = 3.77\text{Å}$. The peak at $0.141q_0$ in the blue line-profile confirms a spatial periodicity of $\sim 2.7\mathrm{nm}$ for the modulated phase along the $[110]$ direction.

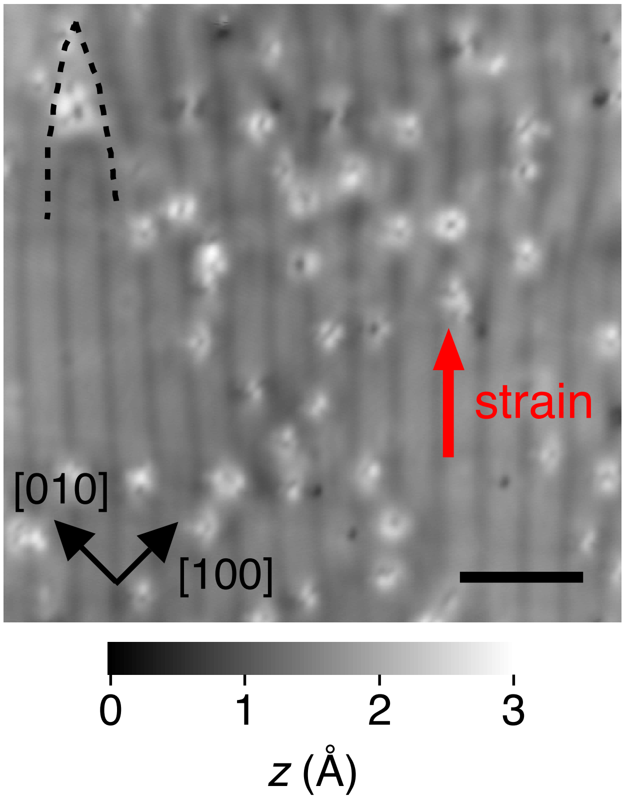

Supplementary Fig. 3: Topological defects on the striped phase. Topographic image of the modulated phase of strained LiFeAs $(40 \times 40\mathrm{nm}^2, 8\mathrm{mV}, 50\mathrm{pA})$. Scale bar: $8\mathrm{nm}$. A red arrow indicates the direction of strain applied. Dotted lines indicate the splitting of a stripe of the modulated phase into two, also deviations of the direction of the stripes from the direction of the strain can be seen in the upper right corner.

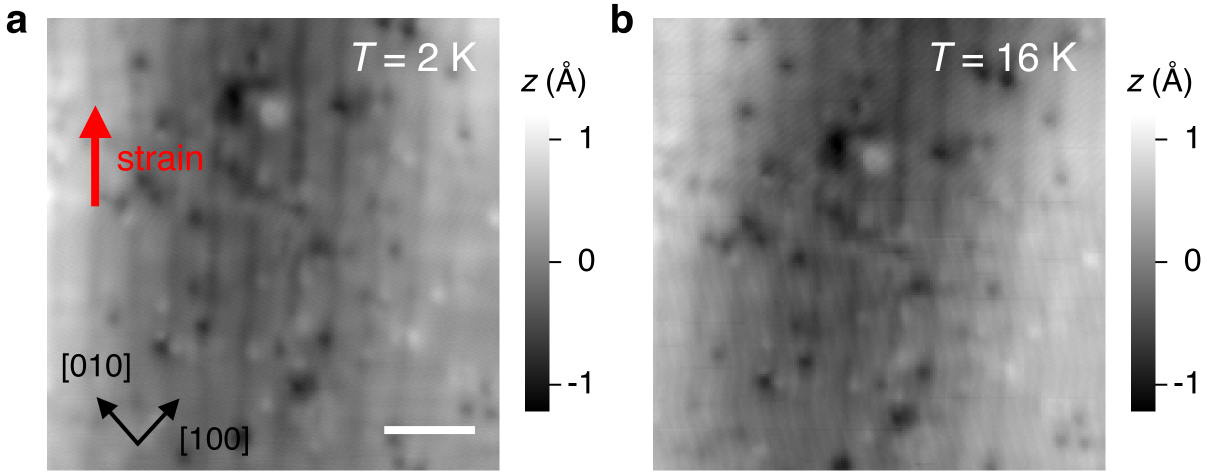

Supplementary Fig. 4: Imaging of the stripe phase at different temperatures. STM topographic images taken at the same position of the modulated phase in the superconducting state at $T = 2\mathrm{K}$ (**a**) and in the normal state at $T = 16\mathrm{K}$ (**b**) $(30 \times 30\mathrm{nm}^2$, $20\mathrm{mV}$, $50\mathrm{pA})$. Scale bar: $6\mathrm{nm}$. The modulated phase persists at temperatures above the superconducting transition temperature T_c. The superconducting transition temperature in the striped phase is $T_\mathrm{c} = 13\mathrm{K}$ (compare Fig. 4a of the main text).

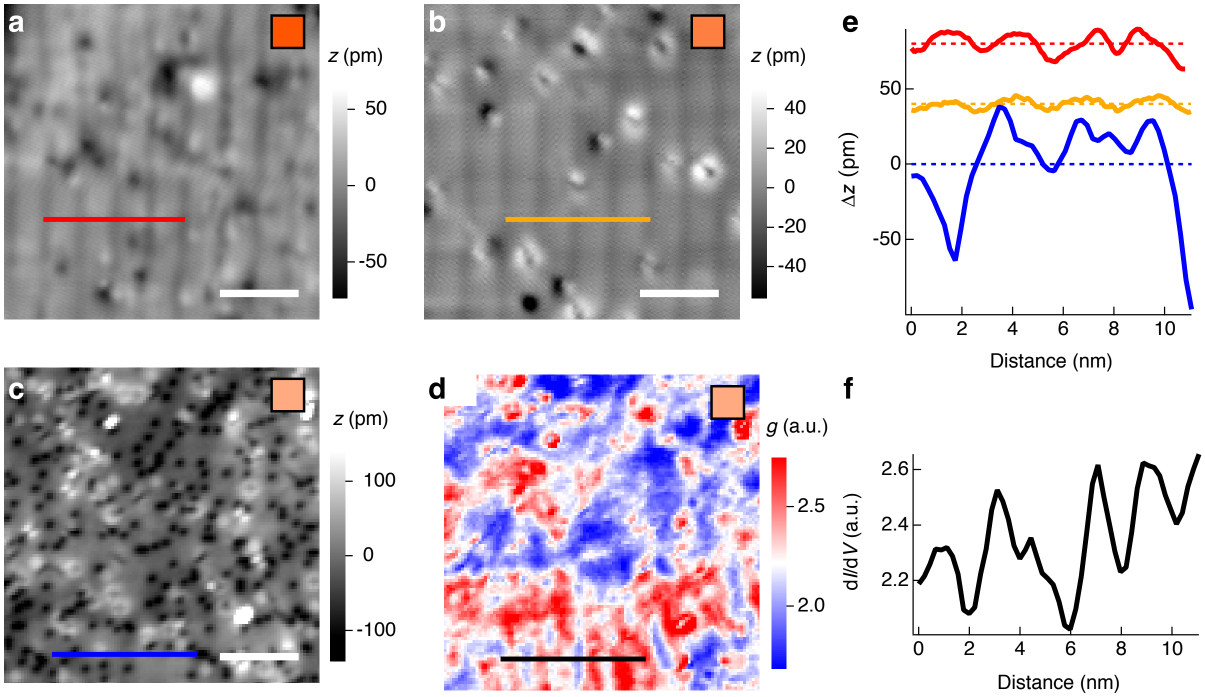

Supplementary Fig. 5: Imaging of the modulated phase with increasing strain. a-c, Topographic images taken in the modulated phase of strained LiFeAs with increasing strain along the $[110]$ direction. Colours of squares in each of the images refer to the corresponding positions in the phase diagram (Fig. 4 in the main text). Image size: **a-c** $(24\mathrm{nm})^2$. Scale bar: **a-c** $6\mathrm{nm}$,. Scanning parameters V_s,I_s: **a** $20\mathrm{mV}$, $50\mathrm{pA}$, **b** $23\mathrm{mV}$, $50\mathrm{pA}$, **c** $20\mathrm{mV}$, $0.4\mathrm{nA}$. **d**, $\mathrm{d}I/\mathrm{d}V$ map at $V = 4\mathrm{mV}$ recorded at the same location as the topographic image in **c**, showing that while the stripes cannot be seen in the topographic image any more at this level of strain, they do show up in the $\mathrm{d}I/\mathrm{d}V$ map. Spectroscopy setpoints: $V_\mathrm{s} = 20\mathrm{mV}$, $I_\mathrm{s} = 0.4\mathrm{nA}$. **e**, Line profiles taken across the modulated phase in **a-c**. Line cuts are offset vertically for clarity, horizontal dashed lines represent $\Delta z = 0\mathrm{pm}$ for each of the line-cuts. **f**, A line-cut taken across the modulated phase in the $\mathrm{d}I/\mathrm{d}V$ map in **d**.

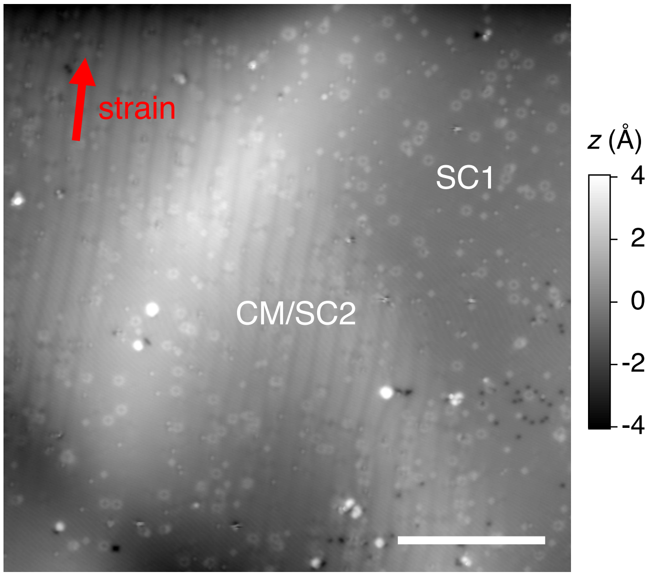

Supplementary Fig. 6: Coexistence of the SC1 and CM/SC2 phases at intermediate strain. Topographic image of the surface of strained LiFeAs crystal at intermediate strain, applied along the [110] direction (indicated by the red arrow). ($77 \times 77\mathrm{nm}^2$, Scale bar = $20\mathrm{nm}$, $V_\mathrm{s} = 30\mathrm{mV}$, $I_\mathrm{s} = 30\mathrm{nA}$).

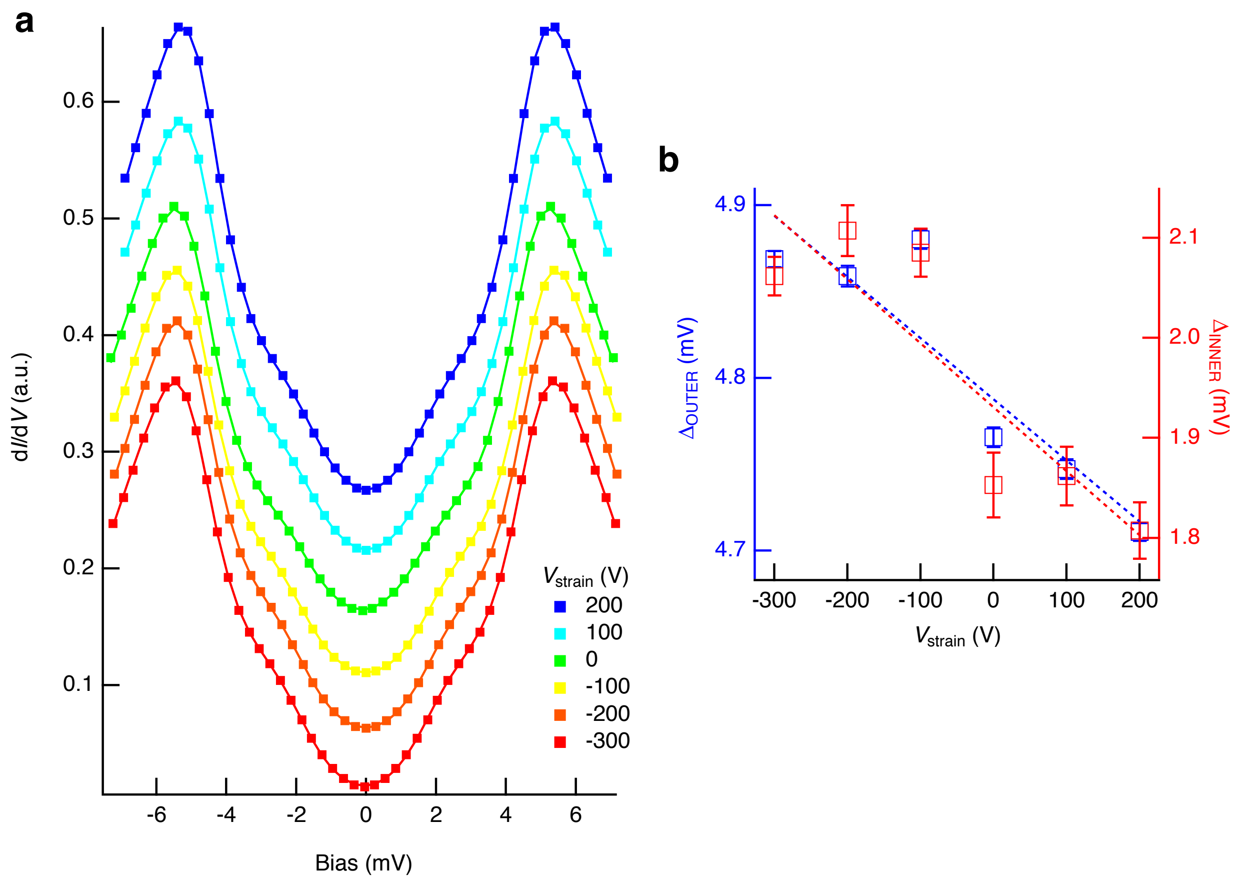

Supplementary Fig. 7: Fitting of the strain-voltage dependent spectra using Dynes equations. a, Symmetrised $\mathrm{d}I/\mathrm{d}V$ spectra obtained at different strain voltage applied along $[100]$, the unsymmetrised data are shown in Fig. 1d. Spectra are vertically offset for clarify. Only the fitted region is shown. Lines are non-linear least squares fits to the symmetrised data using Supplementary Eq. (1). **b**, Scatter plot of the gap size of the outer (blue) and inner (red) superconducting gap in LiFeAs extracted from the fits in **a** versus voltage applied to the piezo stack. Error bars of the gap size are obtained from the non-linear least squares fit and represent the 1σ confidence interval.
